# Fear of rabies and post-bite psychological stress: a qualitative study of YouTube comments from India

**DOI:** 10.1186/s41182-026-01027-0

**Published:** 2026-07-13

**Authors:** Harish Kumar Tiwari, Parimala Mohanty, Arindam Biswas, Jully Gogoi-Tiwari

**Affiliations:** 1 JH CreIndia Foundation, Bhetapara, Guwahati, Assam India; 2 Jyoti and Bhupat Mehta School of Health Science and Technology, Indian Institute of Technology Guwahati, Guwahati, Assam India; 3 DBT-Wellcome Trust India Alliance Intermediate Fellow, Hyderabad, Telangana India; 4 Veterinary Public Health Institute, University of Bern, Bern, Switzerland; 5 School of Veterinary Medicine, College of Environmental and Life Sciences, Murdoch University, Perth, WA Australia

**Keywords:** Rabies, Social media, YouTube, Public perception, Anxiety, India, Thematic analysis

## Abstract

**Background:**

Dog-mediated rabies remains a fatal yet preventable zoonotic disease and continues to cause a substantial proportion of global rabies deaths in India. Although biomedical and epidemiological aspects are well documented, far less attention has been paid to the psychological impact of rabies exposure and the public fear surrounding it.

**Objective:**

To explore how rabies is perceived and emotionally experienced in public online discourse in India, and to characterise patterns of anxiety and distress expressed following dog-bite exposure.

**Methods:**

Public comments (n = 300) were retrieved from six Indian YouTube videos featuring rabies-related content, including news debates, medical explanations, fatal case narratives, free-roaming-dog attack footage, and short videos referencing post-exposure prophylaxis (PEP). Following screening for relevance, 172 comments were analysed using Braun and Clarke’s six-phase inductive thematic analysis framework.

**Results:**

Six principal themes were identified: Rabies Death Anxiety; Medical Anxiety and Reassurance Seeking; Family Protection and Social Impact; Contamination Fear and Avoidance; Hypervigilance; and Contested Attitudes towards Free-Roaming Dogs. Fear-oriented themes predominated. Death anxiety (26%) and medical reassurance seeking (30%) were the most frequent thematic categories. Many comments described persistent rumination, repeated wound-checking, uncertainty regarding vaccine adequacy, and ongoing distress long after exposure. Family-level behavioural changes and avoidance practices were also described. Comments also revealed contrasting views on free-roaming dogs, ranging from support for humane vaccination, sterilisation and coexistence to calls for removal or culling.

**Conclusion:**

The analysed social media discourse suggests that rabies is represented not only as a biomedical threat, but also as a source of persistent uncertainty and fear. Digital comment spaces reveal uncertainty, fear of fatality, and behavioural adaptation following perceived exposure. Integrating psychological considerations and clear risk communication into rabies-control programmes may strengthen public confidence in prevention and treatment strategies.

**Supplementary Information:**

The online version contains supplementary material available at 10.1186/s41182-026-01027-0.

## Introduction

Dog-mediated rabies remains one of the leading causes of human rabies mortality globally, with India accounting for a substantial proportion of reported deaths each year [1]. Despite the availability of effective biomedical interventions, rabies continues to impose a major public health burden in many low- and middle-income countries, including India, where the disease remains endemic [2]. National and global elimination strategies emphasise mass dog vaccination, responsible dog ownership, and sustained community-level advocacy as core pillars for interrupting transmission [3].

While the lethality of untreated rabies renders it uniquely feared, the psychological impact of dog-bite exposure is seldom captured in conventional burden assessments [4]. Beyond the immediate physical injury, bite victims frequently experience prolonged fear, uncertainty, and anticipatory distress related to the possibility of developing rabies [5]. Although such emotional and psychological after-effects of dog bites contribute substantially to individual suffering and perceived disease burden, they are rarely measured in epidemiological surveillance, economic evaluations, or programmatic metrics [6, 7].

Fear following a dog bite often manifests as health anxiety and illness-related uncertainty, characterised by persistent worry about developing fatal consequences, particularly when information is incomplete, ambiguous, or contradictory [6, 8, 9]. Expressions of anxiety surrounding possible rabies exposure may reflect the disease’s variable incubation period, its near-universal fatality once clinical symptoms develop, and public narratives that frequently emphasise mortality more strongly than the preventability of rabies through timely post-exposure care [10]. Misconceptions, misinformation, and culturally mediated beliefs further exacerbate anticipatory fear, creating a context in which psychological distress may be disproportionate to actual biomedical risk [11, 12]. In many cases, the dog bite itself functions as a sentinel event, triggering prolonged anxiety shaped by social discourse, media amplification, and perceived inadequacies of health systems. Interpreting these expressions of fear as responses to uncertainty, structural vulnerability, and culturally specific meanings of rabies is therefore critical [13].

Public narratives of rabies in India frequently conflate biomedical facts, folk beliefs, and media reporting, reinforcing collective anxiety [1, 14]. Regular media coverage of dog bites, coupled with reports of rabies deaths and ubiquitous human–dog interactions in urban and peri-urban areas, sustains a pervasive sense of vulnerability [1, 15]. While One Health and epidemiological research have advanced understanding of rabies transmission, the psychological dimensions including trauma, anticipatory anxiety, and death-related fear remain strikingly underexplored [6].

The uneven access to health care in India that often results in delays or discontinuities in post-bite management, including incomplete adherence to PEP regimens, accentuates this fear [16–18]. Limited awareness of wound care, logistical barriers and uncertainty or misinformation regarding treatment efficacy may contribute to delayed or incomplete post-exposure care. Reports of preventable fatal outcomes may, in turn, intensify anxiety associated with dog bites [1, 19]. Such knowledge gaps, disparities in healthcare access and prevailing misconceptions may generate psychological stress that is partly independent of the underlying biomedical risk. Yet, the prevalence, phenomenology, and social amplification of this fear have rarely been examined empirically.

Digital platforms offer a novel lens to observe these anxieties as they are being increasingly expressed, shared, and reinforced in everyday discourse [20, 21]. The YouTube media platform, in particular, hosts extensive public engagement with health-related content [22], including dog bites and rabies. The user-generated comments to such video uploads reveal spontaneous emotional reactions, coping narratives, and circulating misconceptions [23, 24] related to dog bites and potential rabies infection. These unprompted expressions provide a unique opportunity to analyse and understand collective fear and related anxiety that may not surface in structured surveys or clinical consultations of community members or even bite-victims.

Against this backdrop, we analyse the rabies-related fear and distress articulated on India-related YouTube videos. Specifically, this study aimed to: (i) identify dominant emotional and cognitive themes expressed following exposure to dog-bite or rabies-related content; (ii) interpret these themes through a health-anxiety and trauma-informed lens; and (iii) discuss implications for rabies elimination communication, risk messaging, and psychosocial support for bite victims.

## Materials and methods

This study employed a qualitative digital content analysis of the publicly available YouTube comments related to rabies and dog-bite experiences in India. The aim was not statistical representativeness but the identification of recurring patterns of meaning, consistent with reflexive thematic analysis. The analysis focused on naturally occurring online expressions of fear, uncertainty, reassurance-seeking, and psychosocial responses associated with perceived rabies exposure. Public comments were retrieved from selected India-related YouTube videos in December 2025 and analysed using reflexive thematic analysis, following Braun and Clarke’s six-phase approach [25, 26]. The study was conducted in accordance with ethical guidance for internet-mediated research, given that the data comprised publicly accessible but potentially sensitive user-generated content [27].

To minimise algorithmic personalisation and search bias, two researchers cleared browser histories, cookies, and cached data prior to data collection. Searches were conducted on YouTube using combinations of the terms, "rabies", "dog bite", “dog bite India”, “rabies India”, “rabies vaccine”, “post-exposure prophylaxis”, and "rabies fear/trauma". Search results were screened for relevance to the Indian context, rabies or dog-bite content, public accessibility of comments, and level of viewer engagement. Two authors (AB and PM) independently conducted the search and shortlisted five potentially eligible videos each. Their selections were compared, and any differences were reviewed with the senior authors (HKT and JGT), with the final videos selected through consensus among all four authors. The authors developed a sampling framework based on mutually agreed inclusion and exclusion criteria. Videos were eligible if they: (1) had at least 60 publicly visible viewer comments; (2) originated from India or focused explicitly on the Indian context; (3) depicted, discussed, or reported dog-bite incidents or rabies-related experiences; and (4) contained comments in English or Hindi that were accessible without account login.

Videos were excluded if they were in languages other than English or Hindi, were duplicate uploads, had unavailable or disabled comment sections, or were unrelated to rabies or dog-bite exposure.

Six videos were purposively selected to represent different forms of rabies-related online communication: one national news debate on rabies-control policy; two physician-led educational videos explaining rabies treatment and post-exposure prophylaxis; one news report describing the death of a child from rabies; one CCTV-based news report showing a free-roaming dog attack on a teenage girl; and one short educational video discussing incomplete post-exposure prophylaxis following a dog bite. Although two videos were produced by the same physician, they addressed different post-exposure concerns and generated distinct forms of treatment-related discussion.

From each selected video, the first 50 publicly visible comments displayed under YouTube’s “Newest first” sorting option were extracted, yielding an initial sample of 300 comments. The complete list of selected videos and their URLs is provided in the supplementary material.

### Comment extraction and screening

Comments were extracted verbatim from selected videos. Usernames, timestamps, profile identifiers such as emojis, and hyperlinks were removed to reduce traceability. The individual comment was treated as the primary unit of analysis. Where a comment contained more than one relevant idea, it could receive multiple codes during analysis. Comments were excluded from thematic analysis if they were irrelevant to rabies or dog-bite exposure, nonsensical, promotional, abusive without analytic content, or purely conversational. Screening decisions were made collaboratively by two researchers (AB and PM), with uncertainties resolved through discussion. Comments that expressed fear, uncertainty, treatment-related concerns, perceived exposure, family-level responses, avoidance behaviours, or views on dog management were retained for thematic analysis.

### Coding framework and analytical orientation

The research team had backgrounds in veterinary epidemiology, public health, qualitative research and One Health in India. Throughout the analysis, the team discussed how their prior experience with rabies research could influence interpretation. Before coding, the research team reviewed relevant literature on rabies, dog-bite experiences, health anxiety, risk perception, and online health communication. This helped identify broad areas to look for in the comments, such as fear of rabies, concerns about vaccination, uncertainty about exposure, avoidance of dogs, family-level anxiety, and views on dog management.

Each selected comment was then read carefully and coded according to the relevant meanings it expressed. Some comments expressed more than one concern and were therefore assigned more than one code. For example, a comment could include both fear of death and uncertainty about whether the rabies vaccine schedule was adequate.

In addition to coding the comments, basic information about each video was recorded, including the type of uploader, the format of the video, and the main topic covered. This helped interpret whether certain types of videos, such as medical explanation videos or news reports of rabies deaths, generated different kinds of responses.

Coding was conducted iteratively, and comments could be assigned to more than one code when multiple meanings or sentiments were present within a single entry. Initial coding was undertaken by two researchers, after which interpretations were discussed and iteratively refined through reflexive engagement. Differences were treated as opportunities to examine alternative readings and clarify thematic boundaries rather than as discrepancies requiring statistical agreement. To enhance reflexivity and reduce interpretive bias, analysis was conducted collaboratively to examine and reconcile differing interpretations. Codes were compared and grouped into broader themes through repeated reading and discussion among the researchers. To illustrate the analytic process, an example of coding is provided. For instance, the comment ‘I crossed three streets to avoid a dog; I cannot risk saliva on my shoes or hands’ was initially coded as ‘avoidance behaviour’, ‘fear of contamination’, and ‘perceived exposure risk’. The comment was coded for both contamination-related concern and avoidance behaviour. For the descriptive frequency table, it was assigned to the theme representing its dominant narrative emphasis. The final themes reflected recurring patterns in how commenters expressed fear, uncertainty, reassurance-seeking, family concern, avoidance behaviour, and attitudes towards dogs. The full thematic codebook is presented in supplementary table S1.

Coding and theme development were conducted collaboratively by two researchers (AB and PM) through iterative reading, discussion, and reflexive engagement with the data. Differences in interpretation were explored analytically and refined through discussion with a senior researcher (HKT). Rather than seeking statistical coding consensus, the analysis prioritised interpretive depth, reflexivity, and conceptual coherence.

### Analytic procedure

Data were analysed using Braun and Clarke’s six-phase approach to reflexive thematic analysis [25, 28]. The analysis was mainly inductive, meaning that themes were developed from repeated reading of the comments rather than imposed in advance. At the same time, the researchers remained attentive to concepts relevant to the study, including fear, uncertainty, health anxiety, treatment-related concern, and perceived rabies exposure.

The analysis followed six broad steps. First, all retained comments were read several times to become familiar with the data. Second, relevant parts of the comments were coded according to their main meaning, such as fear of death, uncertainty about vaccination, avoidance of dogs, family concern, or doubts about rabies transmission. Third, similar codes were grouped together to develop preliminary themes. Fourth, these preliminary themes were reviewed against the original comments to check whether they were coherent and clearly distinct from one another. Fifth, the themes were defined and named to reflect the main psychosocial responses identified in the comments. Theme counts were used only to show common patterns in this dataset, not to estimate their prevalence in the wider population. Finally, illustrative quotations were selected and lightly paraphrased to reduce the possibility of online traceability while preserving their original meaning.

### Ethical considerations

Only publicly accessible YouTube comments were analysed, and the researchers did not interact with commenters or collect any private account information. In line with ethical guidance for internet-mediated research, individual informed consent was not sought because the comments were publicly available and the study involved non-interactive analysis of user-generated content [27]. However, the comments were treated as potentially sensitive because they included personal expressions of fear, uncertainty, and distress related to rabies or dog-bite exposure.

To reduce the risk of identification, usernames, timestamps, profile identifiers, and hyperlinks were removed before analysis. Illustrative quotations were lightly paraphrased while preserving their substantive meaning, so that they could not be easily traced through online searches. Findings were analysed and reported at an aggregate thematic level, without attempting to identify or profile individual commenters. The videos were used only as contextual sources from which publicly accessible comments were sampled; no copyrighted video content was reproduced. This study forms part of the broader project, “Implementing a Comprehensive One Health Approach for Rabies Elimination in India”, and received approval from the Institute Human Ethics Committee of IIT Guwahati (IHEC/2024/03).

## Results

**Fig. 1:**
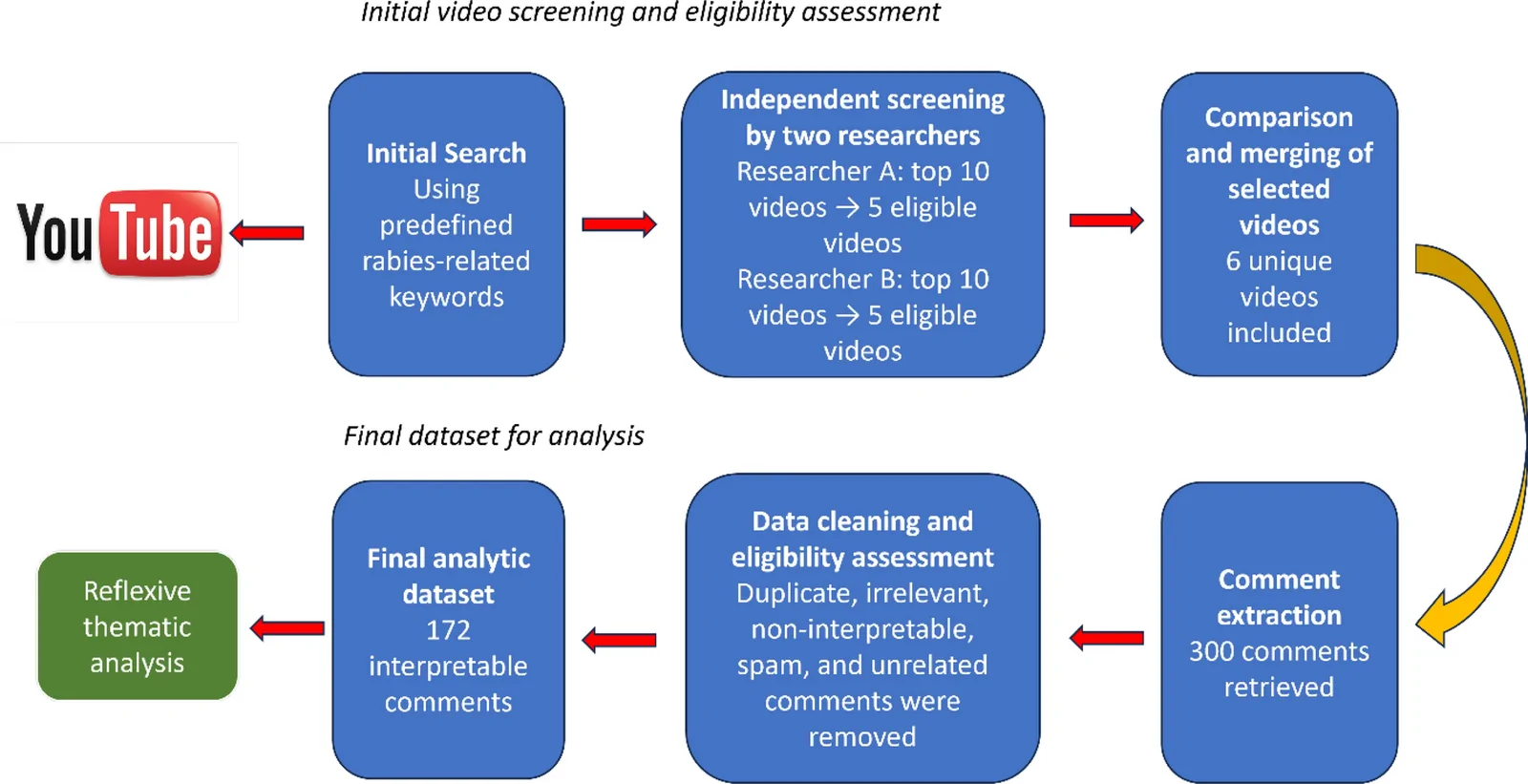
Workflow for YouTube-based comment selection and reflexive thematic analysis

A total of 300 comments posted across six YouTube videos were retrieved and screened for eligibility. Following initial review, 128 comments were excluded because they were irrelevant, uninterpretable, promotional, or unrelated to rabies or dog-bite experiences. The remaining 172 comments contained interpretable content relevant to rabies-related perceptions, experiences, or discussion. The final analytic dataset therefore consisted of these comments that reflected identifiable psychosocial responses associated with perceived rabies exposure, fear, uncertainty, treatment-related concern, or dog-bite experience (Fig. 1).

Six interconnected themes were identified from the final analytic dataset (Table 1). Although these themes are presented separately for clarity, several comments contained overlapping emotional and behavioural elements. For tabulation, each comment was classified according to its dominant narrative emphasis.

**Table 1:** Themes identified in the analysis and their representative meanings (n = 172 comments)

Theme	Frequency (n)	Salient features / representative meanings
Rabies death anxiety	45	Intense fear of dying from rabies; catastrophic thinking; intrusive symptom checking; repeated rumination (“Even after vaccines, I feel doomed; I keep reading about delayed rabies deaths”)
Medical anxiety and reassurance seeking	52	Doubts about adequacy of vaccination; repeated healthcare visits; distrust of medical staff; compulsive online searching for reassurance (“Did five shots really save me?”)
Family protection and social impact	31	Hyper-protective behaviour towards children and relatives; household-level anxiety; social withdrawal; narratives of marital and intergenerational tension
Contamination fear and avoidance	19	Concern about indirect infection through saliva on objects, water, or surfaces; excessive cleaning
Hypervigilance	7	Constant environmental scanning for free-roaming dogs; startle responses; carrying objects for protection; avoidance of dogs and public locations perceived as risky for dog bites
Contested attitudes towards free-roaming dogs	18	Contrasting attitudes towards free-roaming dogs, ranging from support for humane vaccination, sterilisation and coexistence to calls for removal or culling and criticism of animal-welfare advocates

Fear-oriented narratives were the most prominent and were manifested as treatment-related anxiety, reassurance-seeking, and rabies-related death anxiety. Other themes included family-centred protective responses, contamination-related fears, hypervigilant behaviours, and contested attitudes towards dogs. The thematic composition varied across the selected videos (Table 2). Educational and physician-led videos contained a greater proportion of comments involving reassurance-seeking and uncertainty about vaccination, whereas videos depicting dog attacks or rabies deaths elicited stronger fear-based, emotional, and family-centred responses. The psychological constructs used in this study (e.g., health anxiety, hypervigilance, contamination fear) are applied as interpretive analytic categories to describe patterns of meaning in online discourse, and do not represent clinical diagnoses or assessments of individual mental health. The themes focus on expressed perceptions and narratives rather than pathological classification of individuals.

**Table 2:** Distribution of dominant comment themes across the six selected YouTube videos (n = 172 comments)

Theme*	V1	V2	V3	V4	V5	V6	Total (n)	% of total comments
Rabies death anxiety	3	9	9	7	7	10	45	26
Medical anxiety and reassurance seeking	0	21	17	1	1	12	52	30
Family protection and social impact	6	0	0	6	14	5	31	18
Contamination fear and avoidance	0	5	11	1	0	2	19	11
Hypervigilance	2	0	0	0	2	3	7	4
Contested attitudes towards free-roaming dogs	10	0	0	1	5	2	18	11

*Video 1 (V1) National news debate on rabies control policy; Video 2 and 3 (V2 and V3) Physician-led educational video; Video 4 (V4) Fatal case narrative; Video 5 (V5) Dog attack footage, Video 6 (V6) Short video describing consequences of non-adherence to post-exposure prophylaxis following a dog bite

### Rabies death anxiety

Rabies-related death anxiety was expressed following recognised forms of potential exposure, including dog bites and skin-breaking scratches, as well as after non-exposure contact, such as touching or petting dogs, that some commenters nevertheless perceived as carrying a risk of rabies transmission. Many commenters described persistent worry about disease progression, even when the exposure was uncertain or when vaccination had reportedly been completed. Minor scratches, unclear contact with dogs, and uncertainty about saliva exposure often triggered repeated concern about delayed symptom onset.

Several comments suggested that this fear persisted beyond the immediate exposure event. Phrases such as *“years later”, “still thinking about it”,* and *“cannot sleep”* indicated prolonged anxiety and difficulty moving on from the perceived risk. Some commenters also described behaviours consistent with sustained health-related worry, including repeated wound-checking, revisiting rabies-related videos, and searching online for fatality-related information or similar cases.

One commenter stated: *“Even after receiving all recommended vaccines, I cannot stop thinking about whether rabies symptoms might appear years later.”*

Rabies was described in the comments not only as a medical risk, but as a catastrophic and difficult-to-control threat. This fear appeared to be shaped by the perceived fatality of rabies once symptoms develop, even when preventive treatment had reportedly been received.

### Treatment-related anxiety and reassurance-seeking

A frequently observed theme was treatment-related anxiety and reassurance-seeking. Commenters commonly expressed uncertainty about vaccine effectiveness, adequacy of injection schedules, wound management, and whether the treatment they had received was sufficient. Many described their vaccination history in detail and asked whether it provided sufficient protection.

This anxiety was reflected in repeated questions about the number of vaccine doses received, delays in starting treatment, missed or delayed doses, and whether additional booster doses were needed, even after completion of a full course of post-exposure prophylaxis. Other concerns related to the reliability of healthcare services, including vaccine storage, prescription practices, and whether vaccines had been administered according to the correct schedule.

One commenter stated: *“I completed the full vaccination schedule, but I keep wondering if I need more doses. Did the shots really work?”* This illustrates how uncertainty about the effectiveness of post-exposure prophylaxis could persist even after treatment completion. In other cases, anxiety was triggered by repeated exposure before completion of the schedule. For example, one user asked, “I was bitten and I took two vaccines, before I got bitten again. Do I start the schedule all over again?” Such comments suggest that uncertainty was not limited to whether treatment had been initiated, but also involved confusion about how post-exposure prophylaxis should be managed when circumstances changed during the vaccination course.

Several comment threads showed repeated reassurance-seeking, with users returning to clarify details of their exposure or vaccination history and seeking confirmation from peers about the credibility of their post-bite immunisation schedule. In some cases, conflicting responses from other users appeared to intensify concern rather than resolve it. This pattern was particularly visible in comment sections under educational and physician-led videos, where viewers frequently used the platform as an informal space for post-exposure advice and validation.

### Family protection and social impact

Several comments expressed anxiety about protecting family members, particularly children, from potential rabies exposure. Fear was not limited to the individual who had experienced a dog bite, scratch, or possible exposure, but extended to parents, caregivers, and other household members. Commenters described heightened vigilance, especially for children and older family members, including restricting outdoor activities, closely monitoring minor scratches, and altering routine family behaviours to avoid contact with dogs.

One mother described her experience as follows: *“After my child was scratched by a dog, I keep them indoors and constantly monitor for any signs; even minor scratches make me anxious.”* This comment illustrates how a single exposure event could generate continuing fear, leading caregivers to reinterpret ordinary childhood injuries or outdoor activities through the lens of rabies risk.

Some comments also indicated that rabies-related anxiety could generate tension within households, particularly when family members differed in their views about appropriate post-exposure responses. In such cases, fear appeared to influence not only health-seeking decisions but also family communication, caregiving practices, and perceptions of responsibility following a bite or suspected exposure. One commenter sarcastically remarked, “… *(the child is) so scared to even tell his parents that a dog bit him. Nice parenting*”!

In a few instances, concern extended beyond the household and reflected wider social tensions around free-roaming dogs. Comments revealed a visible divide between individuals prioritising child safety and dog-population control, and those emphasising animal protection and cruelty prevention. For example, one commenter attributed child deaths to failures in dog-population management, stating that “*street dog population must be controlled*” and that “*no animal activist will condone the death of the child.*” In contrast, another commenter challenged this framing by arguing that public attention focuses on dog bites but often ignores violence against dogs: “*They see when a dog bit the girl, but when people beat up dogs no one seems to see it.*” These exchanges suggest that rabies-related fear was embedded within a broader contested discourse on public safety, animal welfare, and responsibility for managing free-roaming dog populations.

Overall, these responses suggest that rabies-related anxiety in YouTube comments extended beyond individual concern. It influenced household decision-making, protective behaviours, family routines, and social perceptions of risk.

### Contamination fear and avoidance

A smaller but distinctive subset of comments reflected fears about indirect or environmental transmission of rabies. Commenters expressed concern about possible transmission through saliva on surfaces, water, clothing, shoes, or hands. These concerns were often accompanied by excessive cleaning, avoidance of public spaces, and heightened vigilance around free-roaming dogs.

One commenter described avoiding dogs in everyday movement: *“I crossed three streets to avoid a dog; I cannot risk saliva on my shoes or hands.”*

Some comments reflected contamination-like fears, in which rabies was perceived as an environmental hazard rather than a risk associated mainly with direct exposure through bites, scratches, or mucosal contact with infected saliva. References to *“virus in the air”* or *“droplets from barking dogs”* suggested misinformation about rabies transmission routes, intertwined with genuine distress.

Avoidance behaviours, such as crossing roads to avoid dogs or repeatedly cleaning objects after perceived contact, were described as practical actions rather than hypothetical fears. These comments described behavioural modifications associated with perceived rabies risk; however, the analysis cannot determine whether exposure to the videos preceded or influenced these behaviours.

Although less common than death anxiety or treatment-related reassurance-seeking, these comments show how fear of rabies could extend beyond direct exposure into broader perceptions of environmental threat and contamination.

### Hypervigilance

Hypervigilance-related comments described persistent alertness and behavioural adaptation in response to perceived danger from free-roaming dogs. Commenters reported environmental scanning, heightened startle responses, avoidance of areas where dogs gathered, and carrying sticks or stones for perceived self-protection while walking through public spaces.

One commenter stated: *“I always carry a stick when walking past areas where free-roaming dogs gather; I cannot relax even for a moment.”*

These comments suggest that rabies-related fear was not limited to concern after a bite or scratch, but also shaped everyday movement through public spaces. For some commenters, the presence of free-roaming dogs appeared to produce sustained alertness, anticipatory fear, and changes in walking routes or protective behaviours.

Although numerically limited, this theme reflected a persistent sense of threat in environments where free-roaming dogs were present. It also illustrates how online fear narratives may intersect with everyday experiences of free-roaming dogs to shape risk perception and behaviour.

### Contested attitudes towards free-roaming dogs

Alongside expressions of fear and treatment-related uncertainty, comments also reflected contrasting attitudes towards free-roaming dogs and their management. Some commenters advocated humane approaches to rabies prevention, including vaccination, sterilisation, responsible feeding, and improved population management. These commenters often attributed aggression to human neglect, inadequate vaccination, or poor management rather than to dogs as inherently dangerous.

One commenter argued: “Aggressive behaviour is often linked to human neglect; the focus should be on vaccinating and sterilising dogs rather than killing them.” Others similarly supported coexistence, suggesting that appropriately fed and managed dogs were less likely to pose a threat to communities.

In contrast, some comments expressed anger towards free-roaming dogs and supported their removal, confinement, or killing following bite incidents or reported rabies deaths. These views were sometimes accompanied by criticism of dog feeders, animal-welfare advocates, or “dog lovers”, who were perceived as prioritising animal protection over public safety. For example, one commenter stated that “rabid dogs need to be killed immediately”, while others questioned the role of animal-rights advocates following attacks.

Together, these comments revealed a polarised discourse in which concerns about human safety, rabies prevention, animal welfare, and responsibility for dog-population management were negotiated. Rather than constituting a uniformly compassionate counter-narrative, the theme reflected competing moral and practical positions on how free-roaming dogs should be managed.

### Cross-theme patterns

**Fig. 2:**
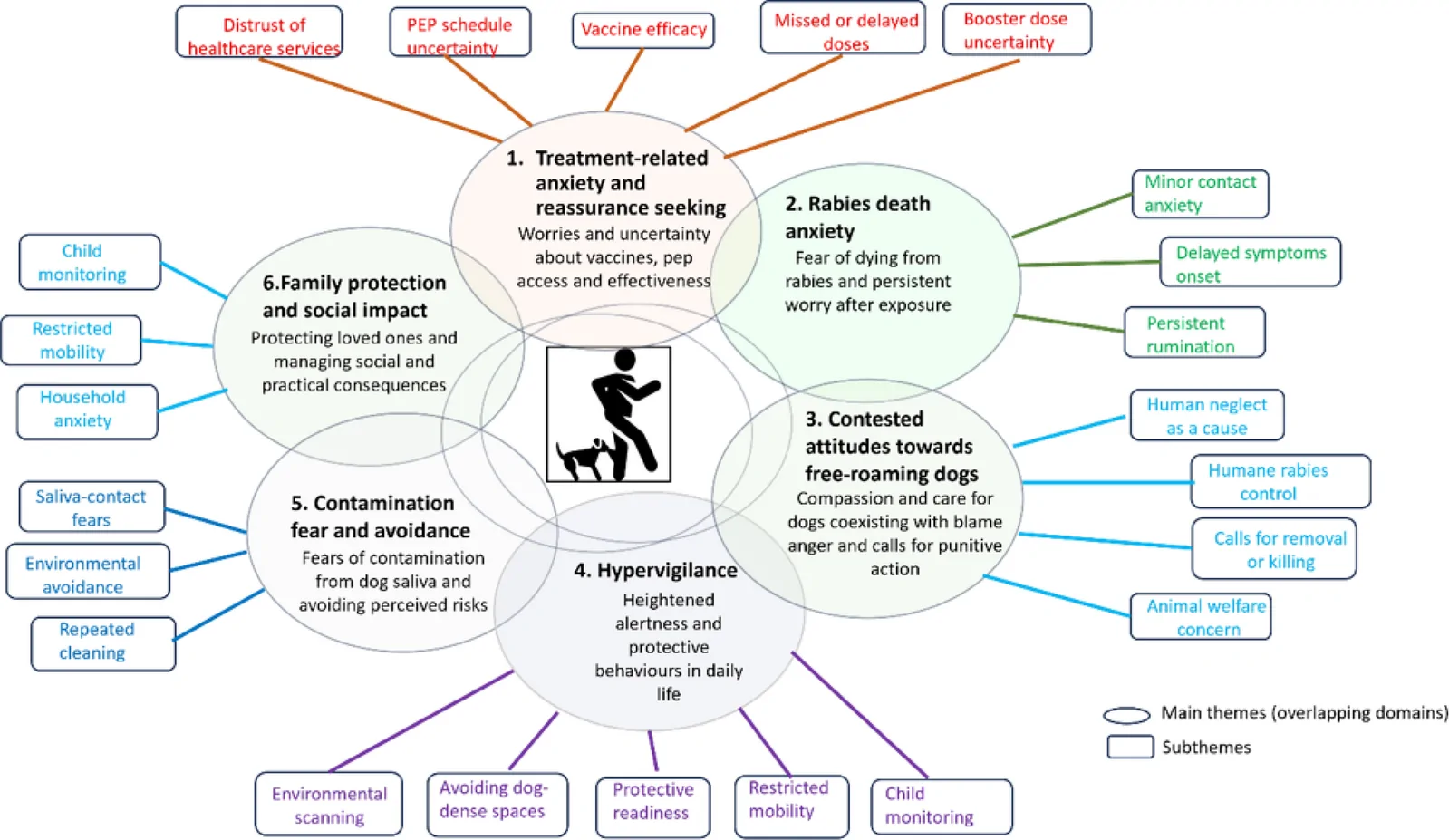
Reflexive thematic map illustrating psychosocial responses associated with rabies-related online discourse in India. The overlapping areas indicate that many comments reflected more than one theme. For example, death anxiety could co-occur with treatment anxiety, or hypervigilance with contamination fear

Across the dataset, comments expressing fear and anxiety substantially outnumbered those advocating humane dog management and rabies-control practices, such as vaccination and sterilisation, as well as comments that were primarily descriptive or informational. Death-related anxiety and treatment-related uncertainty were the dominant narrative currents, often accompanied by repetitive questioning, reassurance-seeking, and behavioural vigilance. Themes were not mutually exclusive; death anxiety frequently co-occurred with reassurance-seeking, while contamination concerns overlapped with hypervigilant behaviours. The theme concerning free-roaming dogs reflected competing positions rather than a uniformly compassionate counter-narrative, including support for humane management and coexistence as well as calls for removal, confinement or culling. A reflexive thematic map is developed to illustrate the psychological responses expressed in rabies-related YouTube comments in India (Fig. 2). The map brings together six interrelated themes that emerged from the analysis: rabies death anxiety, treatment-related anxiety and reassurance-seeking, contamination fear and avoidance, hypervigilance, family protection and social impact, and contested attitudes towards free-roaming dogs. When synthesised together these themes show how rabies-related fear is expressed not only as individual anxiety following actual or perceived exposure, but also highlights a broader social and relational perspective around family protection, household decision-making, everyday movement, and attitudes towards free-roaming dogs.

The illustration show overlap among the themes. Death anxiety was closely linked with treatment-related uncertainty and repeated reassurance-seeking, while contamination fear intersected with avoidance and hypervigilant behaviours. At the social level, concerns about protecting children and other family members coexisted with contested views concerning humane management, public safety and punitive responses towards free-roaming dogs. This study highlights how online comment spaces function as avenues where personal fear, medical uncertainty, social risk perception, and public-health narratives around rabies prevention intersect.

Overall, the comment sections functioned as informal peer-exchange spaces in which personal narratives, uncertainty, and anecdotal experiences circulated rapidly. While a substantial proportion of comments were irrelevant or conversational, the substantive subset revealed persistent and multidimensional psychosocial responses to perceived rabies exposure.

## Discussion

This qualitative analysis of YouTube comments suggests that rabies appears to occupy a distinctive psychological space within the analysed online discourse. Although based on a limited and non-representative digital sample, the recurrence of fear-oriented narratives indicates that rabies is represented not only as an infectious threat but also as a source of profound uncertainty and distress. Expressions of dread, prolonged worry, and repeated reassurance-seeking extended beyond immediate post-bite concerns to include fears about fatality, delayed symptom onset, treatment adequacy and everyday encounters with dogs. In India, where rabies remains an important public health concern, these expressions provide insight into how epidemiological risk is interpreted and emotionally negotiated in online spaces [29, 30].

Fear of rabies-related death emerged as the most emotionally charged theme. Commenters repeatedly described a sense of inevitability using phrases such as “certain death” or “no escape” even when vaccination had been completed. This language reflects the long-standing public perception of rabies as invariably fatal once symptoms develop, a message historically emphasised in prevention campaigns and reinforced through media reporting of individual deaths.

High-profile cases periodically covered in Indian newspapers, particularly those involving children who did not receive timely post-exposure prophylaxis could have reflected this perception [31, 32]. Reports of fatal outcomes despite partial treatment or delayed presentation often circulate widely online, becoming reference points in comment threads [33]. Such narratives may sustain collective memory of rabies as a severe and irreversible disease once clinical symptoms develop, while also portraying possible exposure as a dramatic event requiring urgent but effective preventive action through timely post-exposure prophylaxis [13]. Anthropological accounts have similarly described rabies as a socially charged illness, with visible manifestations of human disease, including hydrophobia, contributing to fear, suffering, and public memory. Hydrophobia is referred to here as a clinical symptom of human rabies and not as a synonym for the disease or as a criterion for recognising rabies in dogs [34, 35]. Within digital spaces, these symbolic dimensions do appear to persist.

The “contagion of fear” described in digital-epidemiology literature provides a useful lens [36, 37]. Algorithmic recommendation systems may shape users’ exposure to emotionally salient health content [38, 39]. However, the present study did not examine individual viewing histories or recommendation pathways and therefore cannot determine whether repeated exposure to rabies-related videos influenced the anxiety expressed in the comments. Reassurance seeking was another of the frequently observed thematic pattern. Many commenters asked about vaccine schedules, queried dosing intervals, and asked whether additional injections were required. This repeated questioning occurred even after completion of recommended PEP regimens. The phenomenon resembles what has been described as cyberchondria, in which online health-information seeking escalates rather than alleviates anxiety [40, 41].

Distrust of public health facilities was also visible in several comments. Concerns about cold-chain maintenance, injection technique, or administrative errors reflect broader debates about health-system reliability. As India’s rabies-control landscape has historically been marked by variability in vaccine availability and uneven communication regarding post-exposure prophylaxis, such anxieties may be rooted in existing gaps in implementation of preventive or control measures [42]. In this context, YouTube comment sections appear to function as informal para-clinical spaces, where individuals seek peer validation in the absence of structured counselling.

The importance of strengthening disease communication at the point of care [43] is also highlighted through these comments. Clear explanation of transmission risk, vaccine efficacy, and symptom timelines delivered empathetically may help reduce the residual uncertainty reflected in the repeated online reassurance seeking. Children may fail to disclose bites or scratches because of fear of reprimand, limited awareness, or the perception that a minor injury is unimportant. Missed or delayed reporting can prevent timely wound care and post-exposure prophylaxis and may therefore contribute to the disproportionate burden of rabies among children, alongside their frequent interaction with dogs, anatomical vulnerability, and dependence on caregivers for treatment-seeking [44].

We suggest integrating brief psychosocial counselling into post-exposure protocols as a feasible addition within existing services [45]. Commenters also described precautions extending beyond the exposed individual to include the household and neighbourhood. Parents reported restricting children’s outdoor activities or closely inspecting minor scratches. In some cases, commenters described tension within families regarding whether adequate medical care had been sought. These accounts suggest that beyond the medical impact, the perceived rabies risk alters daily routines and domestic interactions. Such patterns resemble anticipatory anxiety observed in other public-health emergencies, where fear of recurrence shapes behaviour long after the initial event [46]. In India’s urban settings where free-roaming dogs coexist with dense human populations this dynamic is particularly salient [47]. Periodic reports of dog-bite clusters in cities such as Delhi, Mumbai, and Bengaluru often reignite public debate [48, 49]. For example, widely reported incidents of free-roaming-dog attacks on children in Delhi have led to renewed scrutiny of municipal dog-management policies [50]. In 2022, judicial and municipal discussions in Delhi regarding the relocation or sheltering of aggressive dogs generated substantial public reaction, reflecting tension between animal welfare advocacy and community safety concerns [51]. Introducing risk communication strategies that address families through school-based education, community meetings, and neighbourhood vaccination drives may potentially help transform diffuse anxiety into structured preventive engagement [44, 52].

A subset of comments reflected misunderstanding of transmission routes, including fears of infection through inanimate surfaces or airborne droplets. Although rabies transmission requires direct inoculation of virus-loaded saliva into tissue, uncertainty regarding indirect contact was evident. Such concerns illustrate how partial knowledge may coexist with heightened distress [53]. Avoidance behaviour such as evading localities with high dog density, discarding clothing in contact with dogs, and carrying protection objects such as sticks to keep the dogs away were numerically less common, but these narratives reflect behavioural adaptation shaped by perceived threat. Exposure to media coverage highlighting rabies-risk is also a potential contributor to sustained vigilance. Similar patterns have been documented in other infectious-disease contexts, where intense media focus increases perceived personal susceptibility even when objective risk is low [54, 55]. Public-health messaging therefore needs to balance clarity with reassurance [56]. Overemphasis on fatality without parallel emphasis on vaccine effectiveness may inadvertently reinforce catastrophic thinking. Behaviourally informed communication that validates fear while providing precise information about transmission and prevention could mitigate avoidance behaviours [57].

Notably, some comments articulated empathy towards free-roaming dogs and advocated humane management strategies. These contributors emphasised vaccination, sterilisation, and responsible ownership. Such perspectives are reflective of the ongoing debates surrounding India’s Animal Birth Control (ABC) programme and judicial interventions concerning free-roaming dogs [50].These compassionate narratives may serve a regulatory function within the discourse, and positively frame the responsibility for free-roaming dog management as collective rather than adversarial [58]. Other comments supported removal or killing of free-roaming dogs and criticised dog feeders or animal-welfare advocates. These contrasting responses illustrate how rabies-related fear becomes entangled with contested moral and policy positions concerning public safety and animal welfare. From a One Health perspective, integrating humane dog management with transparent public communication can address both zoonotic control and community wellbeing [59]. Balanced communication that acknowledges legitimate public-safety concerns while promoting evidence-based and humane dog management may reduce antagonistic framing in both physical and digital spaces.

The findings highlight that rabies control in India cannot be conceptualised solely in biomedical terms. The expressions of uncertainty are reflected in YouTube comment discourse, highlighting how digital platforms may serve as spaces where anxieties about rabies exposure are articulated. Rather than implying causality, these patterns should be understood as representations of user perceptions within a publicly available online platform. Persistent fear, uncertainty about treatment adequacy, and community-level tension are psychosocial realities that intersect with prevention efforts. Integrating mental-health awareness into rabies programmes could include brief reassurance protocols in emergency departments, structured discharge counselling after post-exposure prophylaxis, and digital campaigns clarifying vaccine effectiveness and transmission facts.

Such integration supports a broader One Health approach, to rabies elimination by linking human and animal health interventions with psychosocial wellbeing, risk communication and cross-sectoral collaboration [59]. Addressing such psychological dimensions may enhance trust in health services and improve adherence to evidence-based recommendations.

Previous Indian studies have largely focused on knowledge, attitudes, and practices related to rabies or the free-roaming dogs [60, 61]. By contrast, the present analysis presents expressions of how individuals narrate fear, uncertainty, and moral or social tension in real time. Similar emotional dynamics have been documented in digital analyses of other infectious threats, including COVID-19 and HIV, where fear, stigma, and blame circulate rapidly online [62, 63]. The present findings suggest that rabies, despite being an ancient disease, continues to generate comparable affective responses in the digital era. The use of publicly available YouTube comments demonstrates the value of digital qualitative digital content analysis as a complementary method for capturing spontaneous public sentiment. While not representative, such data provide insight into emergent concerns that may not surface in structured surveys.

Overall, the findings highlight the intersection of biomedical risk, psychological uncertainty and contested social responses to rabies in India. The persistence of death anxiety, institutional distrust, family-level behavioural change, and contested narratives about dog management illustrates the complex terrain in which rabies-control policies operate. Recognising and addressing these emotional landscapes may strengthen the effectiveness and public acceptability of elimination strategies.

## Limitations

This study has several limitations. The dataset was restricted to publicly available YouTube comments and therefore cannot be considered representative of the broader Indian population. Commenters’ geographical locations and nationalities could not be verified; therefore, not all comments can be assumed to have originated from users residing in India. Video discovery and comment visibility may also have been influenced by YouTube’s search-ranking and recommendation algorithms, as well as by the use of the ‘Newest first’ comment-sorting option. Selecting the 50 newest comments from each video may have privileged recent discourse and excluded earlier, more highly engaged comment threads. The absence of demographic, clinical, and contextual information also limited interpretive depth, and emotional tone or behavioural responses had to be inferred solely from textual content without follow-up clarification or triangulation. This study included only comments written in English and Hindi and therefore does not capture perspectives expressed in other widely spoken Indian languages, such as Tamil, Bengali, Marathi, Telugu, Assamese, and others. YouTube access and engagement may also be shaped by socioeconomic circumstances, internet availability, digital literacy, age, and patterns of platform use. Consequently, the findings reflect the discourse of a self-selected group of users who viewed and chose to comment on the selected videos, rather than the perspectives of the broader population.

Finally, thematic analysis captures patterns of expression rather than their prevalence or the psychological or clinical status of individual commenters. The findings should therefore be interpreted as discourse-level insights derived from a sampled digital context, rather than as epidemiological estimates or clinical assessments. Despite these limitations, the study provides useful insight into an underexplored psychosocial dimension of rabies by showing how fear, uncertainty, and moral tensions are articulated within contemporary digital spaces.

## Conclusion

Rabies elicited sustained emotional responses within the analysed online discourse, ranging from practical concerns about post-exposure treatment to persistent fear of fatality, uncertainty about vaccine adequacy and repeated reassurance-seeking. These narratives suggest that rabies is represented not only as a biomedical threat but also as a psychologically salient event embedded in social memory, healthcare uncertainty and everyday human–dog interactions.

Attending to these emotional dimensions may strengthen rabies-control efforts. Clear, accessible risk communication; transparent guidance on post-exposure prophylaxis; and humane, community-based dog management strategies could help reduce uncertainty and limit the potential circulation or reinforcement of fear in digital spaces. Integrating psychosocial awareness within One Health-oriented rabies programmes may enhance public trust and improve engagement with prevention and treatment services.

## Supplementary Information

Additional file1 (DOCX 14 KB)

Additional file2 (DOCX 26 KB)

## Data Availability

All data supporting the findings of this study are available within the paper and its Supplementary Information.
