# Supplementary material for: Fear of rabies and post-bite psychological stress: a qualitative study of YouTube comments from India
Source: Trop Med Health. 2026 Jul 13;54:151. doi: 10.1186/s41182-026-01027-0 (PMC13487908; doi:10.1186/s41182-026-01027-0)
Supplement: Supplementary file 1 [file 41182_2026_1027_MOESM1_ESM.docx]

Supplementary file

List of videos included in the study

[Ghaziabad Teen Dies of Rabies A Month After Dog Bite As Virus Went Undetected | The Quint](https://www.youtube.com/watch?v=1j834Cv1BWE)

[Dog Attack | Girl Student Severely Injured In Stray Dog Attack In Indore - YouTube](https://www.youtube.com/watch?v=hP79uVlqPlA)

[Can rabies occur even without a bite? The truth about OCD and vaccines by Dr Anurag Prasad (Major...](https://www.youtube.com/watch?v=2J8Uo3KgvMc)

[Fear of death: Is this OCD or Rabies? Should I get ARV? by Dr Anurag Prasad - YouTube](https://www.youtube.com/watch?v=5Af17Gmbmxs)

[Delhi's Dog Dilemma: SC Intervenes Amid Rabies Fear and Failed Sterilisation - YouTube](https://www.youtube.com/watch?v=Rc_RYsuFLRc)

[He was bitten by a dog twice Rabies Infected Case| Raj Bhosle #shorts #medical #viral](https://www.youtube.com/shorts/W2M2JuE9azU)
