# Supplementary material for: Fear of rabies and post-bite psychological stress: a qualitative study of YouTube comments from India
Source: Trop Med Health. 2026 Jul 13;54:151. doi: 10.1186/s41182-026-01027-0 (PMC13487908; doi:10.1186/s41182-026-01027-0)
Supplement: Supplementary file 2 [file 41182_2026_1027_MOESM2_ESM.docx]

Supplementary Table S1

| **Supplementary Codebook: Thematic Analysis of YouTube Comments on Rabies-Related Fear and Health-Seeking Behaviour in India** | | | | | |
| --- | --- | --- | --- | --- | --- |
| *This codebook presents the codes used in the reflexive thematic analysis. Clinical terms (e.g., health anxiety, cyberchondria, hypervigilance) are used as interpretive lenses, not clinical diagnoses. Colour coding matches the manuscript and analysis spreadsheet. N (%) = frequency of the theme in the analytic dataset (n = 172 comments).* | | | | | |
| **Theme** | **Colour Code / N (%)** | **Theme Definition** | **Code** | **Code Description** | **Illustrative Example from Dataset** |
| **Rabies Death Anxiety** | **Red 45 (26.2%)** | Comments expressing fear of death or fatal outcome specifically linked to rabies. Includes mourning of reported deaths, emotional responses to fatal case narratives, and generalised fatalism about survival after exposure. | **Fear of imminent death from rabies** | Commenter explicitly expresses fear that they or someone they know will die from rabies. | *"Dear sir, I was continuously dying from fear of rabies, but you are like god to me who helped me to live with positivity" (V3) [Note: Interpreted as expression of severe anticipatory death anxiety, not clinical diagnosis.]* |
|  |  |  | **Grief and mourning of rabies fatality** | Comments expressing sadness, condolence, or grief in response to a reported rabies death, particularly of a child. | *"Rip little brother 😭❤️" / "Absolutely heartbreaking" (V5) [Note: Also coded under Social Impact if blame toward parents or system is present.]* |
|  |  |  | **Fatalistic belief about rabies outcome** | Commenter believes rabies is always fatal and expresses hopelessness or resignation. | *"Rabies is 100% fatal. Hence should take the treatment immediately after the bite." (V5) [Note: Factually accurate but coded here when expressed in a fear-laden or fatalistic manner.]* |
|  |  |  | **Delayed treatment fear / guilt** | Commenter expresses anxiety about having delayed vaccination, fearing it may be too late. | *“I was bitten, but I wasn’t vaccinated, its been 13 years now, will there be any problem now?” (V6) [Note: Often co-occurs with Medical Anxiety.]* |
|  |  |  |  |  |  |
| **Medical Anxiety** | **Orange 52 (30.2%)** | Comments reflecting anxiety about medical decisions related to rabies exposure, including uncertainty about vaccination need, dosing, effectiveness, and re-exposure. The largest theme in the dataset. | **Uncertainty about vaccination need after ambiguous exposure** | Commenter is unsure whether a minor or indirect contact (lick, scratch, indirect touch) requires vaccination. | *"Sir before 1 month pet dog lick my legs and don't know there are wound or not and also dog still live then need vaccine or not" (V2) [Note: Core code of this theme; appears across V2, V3, V6.]* |
|  |  |  | **Vaccine dosing and protocol anxiety** | Commenter describes their vaccination schedule in detail and asks whether it was correct or sufficient. | *"Sir a dog bit me on inner elbow it was severe bite and I took Day 0 – Rabishield and intramuscular dose, Day 3 – ID dose... Did I take right or wrong vaccine am I safe?" (V2) [Note: Reflects need for reassurance beyond medical consultation already received.]* |
|  |  |  | **Persistent anxiety after completed vaccination** | Commenter has completed a full vaccination course but continues to feel afraid or seeks additional reassurance. | *"Sir 5 June ko rabies infected very small bite hua tha mene full course of rabies vaccine le liya lekin... abhi mujhe dar lag raha hai bar bar Google search kar rha hu" (V2) [Note: Indicative of features resembling health anxiety or cyberchondria.]* |
|  |  |  | **Distrust of vaccination process or healthcare provider** | Commenter doubts whether the vaccine was correctly administered or whether the healthcare provider was reliable. | *"Sir mujhe vaccine lagane wale staff par hi shak hone lagta hai ki kya pata usne rabies vaccine lagai bhi hai ya nahi" (V2) [Note: Reflects broader healthcare trust deficit.]* |
|  |  |  | **Seeking medical information from YouTube comments** | Commenter directly addresses the video creator or other commenters with specific clinical questions, using YouTube as a substitute for medical consultation. | *"Sir rabies ka incubation period kitna hota hai please reply" (V2) / "If a bat flies over my head and something falls into my eyes, do I need to take an ARV?" (V2) [Note: Highlights YouTube's role as an informal health information platform.]* |
|  |  |  | **OCD-related rabies anxiety** | Commenter explicitly mentions OCD, intrusive thoughts, or an inability to stop seeking reassurance about rabies. | *"Hello sar yah jo sem OCD hai vah mujhe bhi hota hai" (V2) / "Aj mari OCD khatam hova" (V3) [Note: Used as interpretive lens only; not a clinical diagnosis.]* |
|  |  |  |  |  |  |
| **Family, Protection & Social Impact** | **Yellow 31 (18.0%)** | Comments about protecting family members, especially children, from dog bites and rabies; responsibility and blame directed at parents, government, or society; and calls for systemic action on stray dog management. | **Parental anxiety about child's exposure** | Parent (or caregiver) expresses fear about a child's potential rabies exposure and seeks urgent guidance. | *"My son was near a dog. He bitten or not don't know. No blood no scratches no wound. He was wearing jeans. Do I need to give him vaccine. 2 days ago" (V2)  [Note: Reflects anxiety transfer and protective parental response.]* |
|  |  |  | **Blame toward parents for child's death** | Commenter assigns responsibility to the deceased child's parents for the fatal outcome. | *"This is the fault of parents, kids should trust their parents and share everything" (V5) [Note: Also coded under Rabies Death Anxiety if grief is primary.]* |
|  |  |  | **Demand for government or civic action on stray dogs** | Commenter calls for municipal, state, or central government action to address the stray dog menace. | *"If MCD isn't doing its job, why are taxpayers' money still being wasted on same old staff" (V1) / "Every state govt should start a drive and clean the streets" (V5) [Note: Often co-coded with social frustration and anger.]* |
|  |  |  | **Call for mandatory rabies vaccination or public awareness** | Commenter recommends mandatory vaccination, awareness campaigns, or education for children and the public. | *"I think rabies shot must be mandatory for children" (V5) / "Children must be educated and brought awareness of dog bites" (V5)* |
|  |  |  | **Socioeconomic and systemic vulnerability** | Commenter highlights how poverty, illiteracy, or healthcare access shaped the outcome — including lack of awareness in rural or low-income families. | *"Some people accusing the kid's parents... by the look of the boy its almost evident that his parents would be uneducated" (V6)*  *"Our health care system is still at its lowest mindset" (V5) [Note: Reflects structural dimensions of rabies vulnerability.]* |
|  |  |  |  |  |  |
| **Contamination Fear** | **Green 19 (11.0%)** | Comments describing fear of indirect or non-bite transmission — through saliva, shared objects, water, food, or environmental contact. Often involves scenarios that are medically low-risk or non-transmissible. | **Fear of indirect saliva contact** | Commenter fears rabies transmission through indirect routes such as dog saliva on a surface, water, or object. | *"Sir, if the saliva of a rabies dog falls in water and we drink or touch that water, can we get rabies?" (V2)*  *"Sir two days ago a dog licked the tap , and I gargled from the same tap-water, will I get rabies" (V6) [Note: Scenario is medically low-risk; reflects contamination-related fear pattern.]* |
|  |  |  | **Fear of transmission through food or shared items** | Commenter fears contracting rabies by eating food touched by an animal, consuming animal products, or sharing utensils. | *"Sar dog ka jhuta milk pi liye hai to kya rebij vaccine lege" (V3) / "Agr kisi bakri ko kute ne kata ho or us bakri ka meat insan kha le toh kya rabies ho sakta hai?" (V6)* |
|  |  |  | **Environmental contamination fear** | Commenter fears contact with surfaces, soil, grass, or water that an animal may have touched. | *"I fell in a farm... my hand with a cut touched wet grass... wet grass touched my pimple and went into my mouth" (V2)  [Note: Reflects generalised contamination anxiety beyond bite scenarios.]* |
|  |  |  | **Eye/mucous membrane exposure fear** | Commenter fears transmission through the eyes, nose, or mouth from dog barking, splashing, or proximity. | *"Sir mere just bagal mein ek dog bark kiya tha aur uska saliva mere aankh mein chala gaya" (V3) / "If a bat flies over my head and something falls into my eyes" (V2)* |
|  |  |  |  |  |  |
| **Hypervigilance** | **Purple 7 (4.1%)** | Comments describing persistent alertness and behavioural modification in anticipation of dog contact or bite risk. Includes avoidance, heightened scanning of the environment, and inability to function normally due to fear. | **Persistent fear and avoidance behaviour** | Commenter describes ongoing avoidance of dogs, outdoor spaces, or daily activities due to fear of rabies. | *"Dear sir, Mere shoes ya jaha mein bina shoes ke chal raha hota hu or vaha aas paas dog dikh jaye to mujhe rabies ka dar lagne lagta hai" (V2)  [Note: Interpreted as resembling hypervigilance; not clinical diagnosis.]* |
|  |  |  | **Repeated medical help-seeking** | Commenter describes seeking vaccination or medical consultation multiple times for the same exposure or for perceived re-exposures. | *"Main baar baar vaccine lagwata hu or har bar mujhe shak hota hai pata nahi usne lagaya ki nahi lagaya" (V2)  [Note: May co-occur with OCD-related anxiety code under Medical Anxiety.]* |
|  |  |  | **Anxiety months or years after resolved exposure** | Commenter continues to experience fear long after an exposure that was treated or resolved. | *"Sir aaj se lagbhag 5 month pahle... phir bhi dar lagta hai sir please help me" (V2) / "Vaccination ke baad 3 month beet gaye but phir bhi dar lagta hai" (V2)* |
|  |  |  |  |  |  |
| **Dog Protection Attitudes** | **Pink 18 (10.5%)** | Comments reflecting attitudes toward the welfare, rights, or culling of stray dogs. Includes both pro-animal welfare views and anti-stray dog sentiment expressed in response to bite or death incidents. | **Pro-animal welfare / anti-culling stance** | Commenter argues in favour of humane treatment of stray dogs, opposing culling, capture, or removal. | *"Strays are not dangers compare to human" (V1) / "When I go to park I can see both peaceful and aggressive dogs" (V1)  [Note: Sometimes accompanied by critique of dog lovers as disconnected from lived reality of vulnerable communities.]* |
|  |  |  | **Anti-stray dog / pro-removal sentiment** | Commenter expresses anger toward stray dogs or dog activists and supports removal, culling, or sheltering. | *"Shameless dog feeders street dogs are not pets" (V5) / "These rabid dogs need to be instantly killed in India" (V6)  [Note: Often appears in V4 and V5 (dog attack footage; child death news video).]* |
|  |  |  | **Critique of dog activists or 'dog lovers'** | Commenter mocks, challenges, or criticises people who advocate for stray dogs, particularly after an attack. | *"Where are the dog rights activists?" (V5) / "Dog lover kahan mar gaye ab?" (V5)  [Note: Reflects polarised public discourse around stray dog policy.]* |
|  |  |  | **Human-animal coexistence argument** | Commenter argues that humans and stray dogs can coexist if proper feeding, care, or management is provided. | *"We have 20 dogs in my area and I don't have a single bite case. Cos they are fed well and nobody troubles them" (V1)* |
|  |  |  |  |  |  |
| *Abbreviations: V1–V6 = Video 1–6; ARV = Anti-Rabies Vaccine; PEP = Post-Exposure Prophylaxis; OCD = Obsessive-Compulsive Disorder (used as interpretive reference only). Colour codes match Table 1 and the Comment Theme analysis sheet. All examples are paraphrased or reproduced with identifiers removed.* | | | | | |
